# Defying c-Abl signaling circuits through small allosteric compounds

**DOI:** 10.3389/fgene.2014.00392

**Published:** 2014-11-12

**Authors:** Stefania Gonfloni

**Affiliations:** Department of Biology, University of Rome Tor Vergata, Rome, Italy

**Keywords:** c-Abl signaling motifs, stress responses, allosteric compounds

## Abstract

Many extracellular and intracellular signals promote the c-Abl tyrosine kinase activity. c-Abl in turn triggers a multitude of changes either in protein phosphorylation or in gene expression in the cell. Yet, c-Abl takes part in diverse signaling routes because of several domains linked to its catalytic core. Complex conformational changes turn on and off its kinase activity. These changes affect surface features of the c-Abl kinase and likely its capability to bind actin and/or DNA. Two specific inhibitors (ATP-competitive or allosteric compounds) regulate the c-Abl kinase through different mechanisms. NMR studies show that a c-Abl fragment (SH3–SH2-linker–SH1) adopts different conformational states upon binding to each inhibitor. This supports an unconventional use for allosteric compounds to unraveling physiological c-Abl signaling circuits.

## INTRODUCTION

Proteomics has revealed a rather complex picture underlying cellular signaling circuits. Recent studies have indicated an extensive overlap between diverse cellular responses. DNA damage-induced stress response overlaps with pathogen infection response and with heat stress in intact animals (*Caenorhabditis elegans*; Ermolaeva et al., 2013). Signaling connections between DNA damage, stress response, and aging remain elusive in other intact organisms (Gartner and Akay, 2013). Yet, evidence from Ermolaeva et al. (2013) supports a model of an integrated signaling network at the interface of various cell stress routes.

We have discussed about the aberrant c-Abl signaling in the molecular events at the interface of oxidative stress – metabolic regulation, protein aggregation, and DNA damage in neurons (Gonfloni et al., 2012). We have proposed that various stress responses seem to rely on a small set of recurring c-Abl-mediated regulatory circuits (Gonfloni et al., 2012). An emerging theme in neuronal diseases is the aberrant interplay between c-Abl phosphorylation of transcription factors, adaptors, modifiers/enzymes, and ubiquitin-mediated signaling responses (Gonfloni et al., 2012; Ciccone et al., 2013).

In this perspective, I will discuss how modulation of c-Abl, through small molecule allosteric inhibitors/ligands could be exploited to tackle the interface of c-Abl signaling circuits.

## SURFING THE CELL SIGNALING CIRCUITS

Cell metabolism and homeostasis rely on signaling networks of interacting proteins. Posttranslational modifications (PTMs) are crucial for the network. Colocalization of the binding partners converts protein interactions into functional consequences (Kuriyan and Eisenberg, 2007). Crosstalk and interplay between different PTMs give rise to a versatile, rich, and dynamic framework of signaling circuits. Negative (or positive) feedback loops control the amplitude of signaling pathway and the sustained activation in time, conveying signals for irreversible decisions of the cell. A crucial issue for understanding cell signaling is to define how PTMs control changes in metabolism and homeostasis. A simple way is to consider protein domains as basic units of cell signaling (Kuriyan and Cowburn, 1997). Cells may use modular binding motifs like a broad device dedicated to the selective recognition of PTMs (Seet et al., 2006). However, complex biochemical responses can be only achieved in the context of multidomain proteins or multiprotein complexes. Of note, PTMs can also induce a new conformational state that in turn promotes an allosteric regulation of the targeted protein. An example of such an allosteric modulation is the phosphorylation of the activation loop of Src tyrosine kinases. This phosphorylation promotes the productive configuration of the catalytic site. By this mechanism, Src activity (i.e., a productive conformation of the active site in the catalytic domain) also controls the availability of the regulatory domains (SH3-linker; Gonfloni et al., 2000). This effect in turn promotes selective binding/recruitment in multiprotein complexes.

Posttranslational modifications are dynamic and necessary for assembling a temporary platform of local signaling circuits. PTMs work as an allosteric device both for the modified protein and for the assembly/(or reshaping?) of multiprotein complexes. Enzymes are often “switchable,” with their activities controlled by many targets/effectors. The tyrosine kinases are themselves regulated by phosphorylation through various allosteric mechanisms. Non-receptor tyrosine kinases (RTKs) have a conserved catalytic domain (kinase domain = SH1), auto-inhibited by the binding of regulatory domains (SH2, SH3). Such an allosteric regulation links the enzymatic activity of the kinase with the colocalization of its substrate. Allosteric auto-regulation seems to be a recurring feature in cell signaling (Liu and Nussinov, 2013). Protein domains with enzymatic activity (acting as modifiers/writers) are often in tandem with the binding motifs devoted to recognition (acting as readers) of the same modification. This concept is also well substantiated by ubiquitin-mediated signaling. Ubiquitin represents a transferable interaction domain recognized by specialized binding motifs (ubiquitin binding domains, UBDs). Monoubiquitinated proteins often contain a UBD required for their auto-regulation (Seet et al., 2006).

## c-Abl IS AN ALLOSTERIC SIGNALING SWITCH FOR VARIOUS CELL RESPONSES

Non-receptor Abl tyrosine kinases regulate a diverse range of cellular signaling paths. Recent reviews have discussed both the biological functions of the mammalian c-Abl tyrosine kinase (Colicelli, 2010; Wang, 2014) and the role of Abl family kinases in cancer (Greuber et al., 2013). It is beyond the scope of this review discussing these aspects. Interested readers are directed to several excellent reviews on this topic (Sirvent et al., 2008; Hossain et al., 2012; Greuber et al., 2013; Wang, 2014).

Here, I will recall the mechanisms of c-Abl auto-inhibition. The purpose is to highlight the effects of small molecule inhibitors on the conformation of c-Abl.

The Abl kinase family comprises two related proteins Abl1 (c-Abl) and Abl2 (Arg). Both kinases have redundant and unique roles due to their conserved sequence/domain structures. c-Abl and Arg have two different variants (1a and 1b). Both variants are ubiquitously expressed. Abl kinases share a conserved assembly of amino-terminal regulatory and catalytic domain (SH3–SH2-linker–SH1 domain). At the carboxyl terminus region, Abl kinases contain a filamentous (F)-actin-binding domain (ABD; Van Etten et al., 1994). c-Abl and Arg are less conserved in the middle region. So, Arg lacks of the three nuclear localization signal (NLS) motifs and localizes in the cytoplasm and in cell periphery (Miller et al., 2004). By contrast, c-Abl is present in the cytosol but also in organelles, such as the endoplasmic reticulum (ER) the mitochondria (Ito et al., 2001), or the nucleus (Wen et al., 1996). The diverse localization of c-Abl is modulated by PTMs (Yoshida et al., 2005). The formation of distinct multiprotein complexes is likely regulated by a dynamic spatial recruitment. Spatial distribution of c-Abl is linked to the catalytic competence of the kinase. The latter is in constant equilibrium between low (fully inhibited) and high (fully activated) levels of activity (Hantschel and Superti-Furga, 2004). c-Abl signaling is not only dependent from the outcomes derived from its enzymatic kinase activity. But, it depends from dynamic recruitment of c-Abl into different protein complexes (and subcellular compartments). In vertebrates, its C-terminal F-ABD mediates actin binding, bundling and microtubule crosslinking (Bradley and Koleske, 2009). This has important consequences for cell adhesion, migration (Woodring et al., 2003), intracellular trafficking (Rotty et al., 2013), endocytosis (Lonskaya et al., 2013), autophagy (Yogalingam and Pendergast, 2008; Hebron et al., 2013b; Lonskaya et al., 2013). In *Drosophila*, D-abl signaling is linked to actin dynamics and cell adhesion (reviewed by Lanier and Gertler, 2000; Hernandez et al., 2004). Recent evidence indicates that the D-abl kinase signaling regulates the Golgi complex architecture in neurons (Kannan et al., 2014). These data suggest that some of the effects of c-Abl signaling may arise from alterations of protein trafficking and secretion (Kannan et al., 2014). Kinase-independent functions of c-Abl have been already described (Henkemeyer et al., 1990; Chen et al., 2006). Evidence supports a kinase-independent function of *Drosophila* Abl for axonal guidance outcomes (O’Donnell and Bashaw, 2013). These results are consistent with a model for stepwise scaffolding and kinase functions of Abl in cell motility (Lapetina et al., 2009). Likely in a stepwise manner c-Abl promotes (or prevents) the formation of diverse signaling platforms within the cell. Specific outcomes rely on the full catalytic competence of the Abl kinase. The latter is due to local enrichment and/or a concomitant allosteric binding/removal of activators/adaptors/co-inhibitors (as it occurs in the nucleus during apoptosis). Both local enrichment and expression/localization of binding partners (adaptors/co-inhibitors) depend from cellular context.

## ALLOSTERIC REGULATION OF c-Abl

**FIGURE 1:**
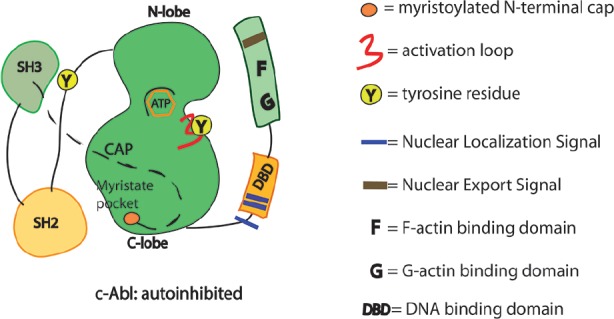
**Schematics of the functional domains in c-Abl.** SH3, Src homology 3 domain: SH2, Src homology 2 domain. Y245 is in the linker region connecting SH2 domain with the kinase domain. Y412 is in the activation loop of the kinase domain. Phosphorylation in these two sites may prevent the auto-inhibited conformation of the c-Abl.

The auto-inhibited conformation of c-Abl is controlled through SH3–SH2-linker unit as in the Src family tyrosine kinases. In c-Src, the SH2 domain interacts with the C-terminal tail phosphotyrosine residue (Y527). By contrast, in c-Abl, the SH2 domain interacts more intimately with the large C-terminal lobe of the kinase domain (SH1). Interestingly, the tight interactions of the SH2–SH1 domain are induced by the binding of the myristoylated residues of the N-terminal region into a hydrophobic pocket of the kinase (Figure 1; Nagar et al., 2003). c-Abl requires the N-terminal myristoyl group (only present in Abl1b variant) to help the proper SH3–SH2-linker docking and inhibition (Iacob et al., 2011; Corbi-Verge et al., 2013; de Oliveira et al., 2013). Allosteric inhibitory interactions for the Abl1a variant are still poorly understood. Such interactions likely involve the binding of other inhibitors/adaptors. Small molecule compounds (GNF-2 and GNF-5) targeting the myristate pocket in the C-lobe of the kinase domain do act as allosteric c-Abl inhibitors (Adrian et al., 2006; Fabbro et al., 2010; Zhang et al., 2010). The relevance of the myristoyl-binding pocket is further reinforced by the recent discovery of small-molecule c-Abl activators that dock into the same site (Yang et al., 2011; Hong et al., 2014). Upon c-Abl activation and removal of the allosteric interactions, the SH2 domain interacts with the N-terminal lobe of the kinase domain by using different surfaces of the SH2 domain (Hantschel, 2012). Compelling evidence indicates that the SH2 domain acts as a positive allosteric activator via the formation of an internal interface with the N-terminal lobe of kinase domain (Hantschel, 2012). Of note, the positioning of the SH2 domain facilitates multisite phosphorylation of substrates by c-Abl (Filippakopoulos et al., 2008; Grebien et al., 2011). However, alternative active states of c-Abl that do not require the SH2/kinase interface to function may occur when local clustering of c-Abl kinase core is sufficient for triggering transphosphorylation of the activation loop. In these circumstances the SH2 domain displacement from the back of the kinase domain is dispensable (Panjarian et al., 2013a,b). In short, the multidomain kinases like c-Abl can assume various conformational states and take more than one path to activation.

## EMERGING CONCEPTS FROM THE SOLUTION CONFORMATIONS OF c-Abl

Recent structural studies using NMR in combination with small angle X-ray scattering (SAXS) of a c-Abl fragment (SH3–SH2-linker–SH1 domains) provide the first structural information of apo form of c-Abl in the absence of small molecule inhibitors (Skora et al., 2013). The apo form of c-abl adopts the “closed” conformation with the SH3–SH2 regulatory unit engaged with the kinase domain. However, addition of Imatinib (an ATP-competitive inhibitor) induces both a large structural rearrangement of the kinase domain and the detachment of the SH3–SH2 regulatory unit from the kinase domain leading to the formation of an “open” inactive state, where the ATP binding site is not accessible. In contrast to Imatinib, addition of the myristoyl pocket ligand GNF-5 to apo c-Abl induces only limited local changes around the myristoyl-binding pocket and keeps the SH3–SH2 regulatory unit in the “closed” state. Addition of GNF-5 to the “open” inactive state (c-Abl in complex with Imatinib) restores the “closed” inactive conformation (Skora et al., 2013). Under physiological conditions the “open” and “closed” conformations of c-Abl may be in equilibrium, which can be altered by the presence of specific inhibitors (ATP-competitive and/or allosteric ones).

It has been proposed that the ABD may stabilize the auto-inhibited conformation of the kinase by binding to F-actin (Woodring et al., 2003). Interestingly, the inhibitory effect of F-actin requires the SH2-kinase domain interaction to maintain the auto-inhibited conformation (Woodring et al., 2003). Small molecule inhibitors may induce a structural remodeling of the auto-inhibited conformation. This in turn may perturb domain interactions and consequently impinge on adaptor/effector/substrate binding, modulating c-Abl signaling dynamics. Cells treated with Imatinib show a profound change in the shape and a more rapid migration when plated on collagen-coated substrates (Chen et al., 2013). GNF-2 promotes a translocation of c-Abl to the endoplasmic reticulum (Choi et al., 2009). It is tempting to consider that GNF-2 promotes a dynamic recruitment of c-Abl into different subcellular compartments and protein contexts. A timely relocalization of c-Abl/GNF-2 complex in a specific subcellular compartment may induce *per se* a signaling circuitry.

An unproductive conformation (“open” inactive state) of the catalytic site induced by Imatinib may promote the availability of the regulatory domains (SH3–SH2-linker–ABD) with profound effects on c-Abl-interactome.

Molecular switches like c-Abl have modular domains required for their assembly into multiprotein complexes. Yet, c-Abl has also a modifier/kinase domain to regulate scaffold/signaling dynamics. The challenge now is to understand how such a complex signaling assembly is regulated in time and space (Hossain et al., 2012). Of note, both local enrichment and expression/localization of binding partners (adaptors/co-inhibitors) are dictated from the cell type and signaling context.

## ABERRANT c-Abl SIGNALING

The c-Abl kinase was early discovered as the oncogene in the Abelson murine leukemia virus (Goff et al., 1980) and then associated with human leukemias (Ben-Neriah et al., 1986). Several reports have indicated that c-Abl is a substrate and an activator of RTK. This bidirectional activation contributes to robust and persistent RTK signaling (Bromann et al., 2004). Cancer cells, expressing high levels of c-Abl, become dependent from its catalytic activity for growth and viability (reviewed in Greuber et al., 2013). On the other hand, in neurons aberrant c-Abl activation causes hyperphosphorylation, misfolding, and protein aggregation of tau protein, or alpha-synuclein. Such effects are considered hallmarks of neurodegenerative diseases (Ciccone et al., 2013; Tenreiro et al., 2014).

## c-Abl SIGNALING MEETS UBIQUITIN-MEDIATED RESPONSE

Cell signaling relies on PTMs for its regulation. The interplay and the crosstalk between phosphorylation and ubiquitination represent a recurrent theme in cell signaling (Hunter, 2007). We have discussed about some connections occurring between c-Abl phosphorylation and ubiquitin-mediated signaling in DNA damage response (Maiani et al., 2011). Kinase phosphorylation/activation often triggers ubiquitination. Activated forms of c-Abl are more unstable than wild-type (Echarri and Pendergast, 2001).

Compelling evidence indicates that c-Abl modulates the degradation of two proteins implicated in the pathogenesis of Parkinson’s disease (Mahul-Mellier et al., 2014). A specific inhibitor of c-Abl like Nilotinib, used for leukemia treatment, promotes autophagic degradation of α-synuclein while protecting neurons (Hebron et al., 2013a; Lonskaya et al., 2014). Interestingly, Nilotinib-induced autophagic changes increase endogenous Parkin level and ubiquitination, favoring amyloid clearance (Lonskaya et al., 2014). Taken together the data indicate that small molecule c-Abl inhibitors (ATP-competitive) may modulate the interplay between c-Abl and ubiquitin-mediated signaling. Convincing evidence indicates that c-Abl-mediated phosphorylation directly regulates the activity of some substrate E3 ligases (Zuckerman et al., 2009; Chan et al., 2013). In addition, negative regulation of E3 ligase activity by c-Abl can require a specific recruitment of c-Abl into a complex with adaptor molecules, necessary for proper localization (Skouloudaki and Walz, 2012). Of note, tyrosine phosphorylation of Parkin on Y143 inhibits its E3 ligase activity leading to an accumulation of Parkin substrates (Ko et al., 2010). On the contrary, Imatinib treatment restores the E3 ligase activity of Parkin and its protective function. Interestingly, administration of Nilotinib has reduced the c-Abl activation and the levels of the Parkin substrate (PARIS) without preventing tyrosine phosphorylation of Parkin and accumulation of the Parkin substrate AIMP2. This suggests that the protective effect of Nilotinib may be in part Parkin-independent or related to the pharmacodynamics properties of Nilotinib (Karuppagounder et al., 2014). Of note, Nilotinib belongs to a second generation of inhibitor, ATP-competitive like Imatinib. It is more potent (>20-fold), exhibiting activity toward the majority of Imatinib-resistant mutations. The majority of Nilotinib–c-Abl interactions overlap with those described in the Imatinib–c-Abl complex. However, Nilotinib binds many of mutants resistant to Imatinib and is less sensitive to mutations of the C-lobe of the kinase (Reddy and Aggarwal, 2012). This may reflect that Nilotinib induces a dynamic rearrangement in the catalytic domain, attenuating the effects of mutations on the auto-inhibited conformation.

## CONCLUSION

Compelling evidence indicates the physiological relevance of the interface between c-Abl signaling and stress response, metabolic regulation mediated by transcription factors (Gonfloni et al., 2012). A small molecule that binds to the myristate binding pocket in the C-lobe of the Abl kinase was shown to inhibit Bcr-Abl (Zhang et al., 2010). This result indicates a functional connection between the myristate pocket and the kinase active site. Data from hydrogen exchange mass spectrometry indicate that binding of GNF-2/GNF-5 induces a dynamic conformation of residues near Thr315 that allows ATP-competitive inhibitors to tolerate the isoleucine at this position (Zhang et al., 2010). However, it remains still elusive how changes in the myristate pocket are communicated to the ATP binding site of the kinase. Molecular dynamic (MD) simulations (Fallacara et al., 2014) and emerging evidence from recent structural studies using NMR indicate that c-Abl may assume different conformational states in presence with different small molecule inhibitors (Skora et al., 2013). Together these data indicate that GNF-2 binding induces a more compact conformation of SH2-kinase domain interface of c-Abl. Therefore allosteric ligands for myristoyl pocket may be valuable tools for tackling the interface of c-Abl signaling. They could represent a way to attenuate the enzymatic activity while impinging on critical SH2 domain interactions. This in turn may rewire downstream signaling circuits and/or pathways. Recent evidence on the use of GNF-2 *in vivo* supports such a model (Maiani et al., 2012). c-Abl interacts with a large number of proteins (up to now more than 100) most of them are also c-Abl substrates (85%; Colicelli, 2010), in line with the idea that substrates can work as allosteric activators of the kinase. A better understanding of the spatiotemporal regulation of c-Abl signaling may allow us to modulate c-Abl signaling into specific subcellular compartments, with important consequences for cell homeostasis. These studies will take advantage from small allosteric compounds/activators as tools to investigate the biological functions of c-Abl (Hong et al., 2014). Surely, a detailed understanding of c-Abl functions will help to develop combined targeted therapies in order to rewire the physiological regulatory circuits in cancer cells and in aged neurons.

### Conflict of Interest Statement

The author declares that the research was conducted in the absence of any commercial or financial relationships that could be construed as a potential conflict of interest.
